# Metropolitan Hospital Sunday Fund, 1896

**Published:** 1896-08-01

**Authors:** 


### METROPOLITAN HOSPITAL SUNDAY FUND, 1896.

### PARTICULARS OF THE AWARDS.

The following is the report of the Committee of Distribution adopted by the Council on the 28th ult., Sir Sydney H. Waterlow in the chair:—

Your committee have the honour to recommend awards to 180 of the institutions enumerated in the appendix to this report, being an increase of 75 since the first awards were made in 1873. The total amount available for distribution, after allowing for liabilities and the usual current expenses, is £44,029. Of this total £41,829 is now recommended to 125 hospitals and 55 dispensaries. Five per cent. of the total collected—viz, about £2,200—is set apart to purchase surgical appliances (in obedience to Law XII.), in monthly proportions during the ensuing year.

Your committee recommend that all payments to the Fund after this date be carried to the credit of next year's Fund.

In compliance with an order of the Council, and for the special use of its members, tables of statistics have been prepared as usual, showing an analysis of the number of beds in hospitals, the cost of patients both at hospitals and dispensaries, the proportionate expense of management, as well as other valuable information, and copies are sent to every member of the Council.

Your committee have this year had occasion to question the administration of nineteen institutions. Each of these have been given the opportunity of sending a deputation in conference. Your committee are, however, glad to report that in most cases the objections raised were frequently explained satisfactorily. There are now very few institutions which do not publish their accounts on the uniform system agreed upon. One special hospital, having no present need, gets no award. One convalescent home where the charge for maintenance of patients was deemed excessive, has no award recommended. The managers of an institution, classed as a dispensary, were requested by your committee to produce their minute-book. This they declined to do, and your committee, being doubtful of the existence of any such book, have recommended no award. The result of all their interviews will, your committee hope, lead to improvement in the future.

Awards are therefore recommended to 161 institutions, on full bases, to sixteen on slightly reduced bases. The remaining three applicants have no awards recommended.

#### Twenty-six General Hospitals.

Charing Cross Hospital, £1,054; French Hospital, £383; German Hospital, £479; Great Northern Central Hospital, £479; Guy's Hospital, £814; Hampstead Hospital, £115; Italian Hospital, £71; King's College Hospital, £1,581; London Hospital, £3,833; London Homœopathic Hospital, £162; Phillips Memorial Homœopathic Hospital, £33; London Temperance Hospital, £699; Metropolitan Hospital, £479; Miller Hospital and Royal Kent Dispensary, £263; North-West London Hospital, £383; Poplar Hospital, £440; Royal Free Hospital, £910; St. George's Hospital, £1,437; SS. John and Elizabeth Hospital, £95; St. Mary's Hospital, £1,916; Seamen's Hospital Society, £958; The Middlesex Hospital, £2,204; University College Hospital, £1,341; West Ham Hospital, £287; West London Hospital, £575; Westminster Hospital, £1,150.

#### Special Hospitals.

Five Chest Hospitals.—City of London Hospital for Diseases of the Chest, Victoria Park, £1,006; Hospital for Consumption, Brompton, £1,542; North London Consumption Hospital, Hampstead, £345; Royal Hospital for Diseases of the Chest, City Road, £383; Royal National Hospital for Consumption (Ventnor), £287.

Fourteen Children's Hospitals.—Alexandra Hospital for Hip Disease, W.C., £172; Barnet Home Hospital, £33; Belgrave Hospital for Children, S.W., £143; Cheyne Hospital for Incurable Children, S.W., £143; East London Hospital for Children, Shadwell, E., £431; Evelina Hospital for Sick Children, Southwark, S.E., £431; Home for Incurable Children, Maida Vale, W., £67; Home for Sick Children, Sydenham, S.E., £143; Hospital for Sick Children, Great Ormond Street, W.C., £795; North-Eastern Hospital for Children, Hackney Road, N.E., £287; Paddington Green Hospital for Children, W., £143; Victoria Hospital for Children, King's Road, Chelsea, S.W., £575; Victoria Home, Margate, £28; "The Vine," Sevenoaks, £28.

Six Lying-in Hospitals.—British Lying-in Hospital, Endell Street, W.C., £38; City of London Lying-in Hospital, City Road, E.C., £71; Clapham Maternity Hospital, £19; East End Mother's Home, £67; General Lying-in Hospital, Lambeth, S.E., £57; Queen Charlotte's Lying-in Hospital, Marylebone Road, W., £287.

Six Hospitals for Women.—Chelsea Hospital for Women, S.W., £237; Hospital for Women, Soho Square, W., £412; Grosvenor Hospital for Women and Children, Vincent Square, S.W., £105; New Hospital for Women, Euston Road, W.C., £201; Royal Hospital for Children and Women, Lambeth, S.E., £220; Samaritan Free Hospital, Lower Seymour Street, W., £527.

#### Twenty-six other Special Hospitals.

Cancer Hospital, Brompton, S.W., £575; London Fever Hospital, Islington, N., £728; Gordon Hospital for Fistula, Vauxhall Bridge Road, S.W., £38; St. Mark's Hospital for Fistula, City Road, E.C., £38; National Hospital for the Diseases of the Heart and Paralysis, Soho Square, W., £57; Female Lock Hospital, Harrow Road, W., £153; Male Lock Hospital, Soho, W., nil; Hospital for Epilepsy, Paralysis, and other Diseases of the Nervous System, Regent's Park, N.W., £57; National Hospital for the Paralyzed and Epileptic, W.C., £824; West-End Hospital for Diseases of the Nervous System, £105; Central London Ophthalmic Hospital, Gray's Inn Road, W.C., £32; Royal Eye Hospital, St. George's Circus, S.E., £182; Royal London Ophthalmic Hospital, Moorfields, E.C., £536; Royal Westminster Ophthalmic Hospital, Charing Cross, W.C., £95; Western Ophthalmic Hospital, Marylebone Road, W., £44; City Orthopædic Hospital, Hatton Garden, E.C., £76; National Orthopædic Hospital, Great Portland Street, W., £62; Royal Orthopædic Hospital, Oxford Street, W., £76; Royal Sea-Bathing Infirmary, Margate, £335; Hospital for Diseases of the Skin, Stamford Street, S.E., £9; St. Peter's Hospital for Stone, Covent Garden, £28; Central London Throat and Ear Hospital, Gray's Inn Road, W.C., £47; Hospital for Diseases of the Throat, Golden Square, W.C., £28; Royal Ear Hospital, Frith Street, W., £5; Dental Hospital of London, Leicester Square, W.C., £124; National Dental Hospital, 149, Great Portland Street, W., £110.

#### Twenty-three Convalescent Hospitals.

Metropolitan Convalescent Institution, Sackville Street, W., £622; Bexhill Convalescent Home, Sackville Street, W., £387; All Saints' Convalescent Hospital, Eastbourne, £527; Mrs. Gladstone's Convalescent Home, Woodford, £95; Hahnemann Convalescent Home, Bournemouth, £38; Herbert Convalescent Home, Bournemouth, £28; Herne Bay Baldwin-Brown Convalescent Home, £23; Herne Bay Mothers' Home, £38; Homœopathic Convalescent Home, Eastbourne, £9; King's College Hospital Convalescent Home, Hemel Hempstead, £81; Mrs. Kitto's Convalescent Home, Reigate, £86; London and Brighton Female Convalescent Home, £38; Mary Wardell Convalescent Home for Scarlet Fever, £101; Morley House Convalescent Home for Working Men, £143; Princess Frederica's Convalescent Home, East Molesey, nil; St. Andrew's Convalescent Hospital, Clewer, £172; St. Andrew's Convalescent Home, Folkestone, £249; St. John's Home for Convalescent and Crippled Children, Brighton, £67; St. Joseph's Convalescent Home, Bournemouth, £52; St. Leonards-on-Sea Convalescent Home for Poor Children, £105; St. Michael's Convalescent Home, Westgate-on-Sea, £38; Seaside Convalescent Hospital, Seaford, £134; Warley Convalescent Cottage Hospital, Essex, £7.

#### Twelve Cottage Hospitals.

Beckenham Cottage Hospital, £52; Blackheath and Charlton Cottage Hospital, £67; Bromley, Kent, Cottage Hospital, £79; Chislehurst, Sidcup, and Cray Valley Cottage Hospital, 43; Eltham Cottage Hospital, £31; Enfield Cottage Hospital, £33; Epsom and Ewell Cottage Hospital, £55; Hounslow Cottage Hospital, £23; Reigate and Redhill Cottage Hospital, £52; Sidcup Cottage Hospital, £31; Wimbledon Cottage Hospital, £38; Woolwich and Plumstead Cottage Hospital, £29.

#### Seven Institutions.

Establishment for Gentlewomen, Harley Street, £105; S. Saviour's Hospital and Nursing Home, £47; National Sanatorium for Consumption, Bournemouth, £57; Invalid Asylum, Stoke Newington, £52; Firs Home, Bournemouth, £38; Friedenheim Home for the Dying, £191; St. Catherine's Home, Ventnor, £28.

Fifty five Dispensaries.

Battersea Provident Dispensary, £67; Bloomsbury Provident Dispensary, £11; Brixton, &c., Dispensary, £57; Brompton Provident Dispensary, £25; Camberwell Provident Dispensary, £95; Camden Provident Dispensary, £13; Chelsea, Brompton, and Belgrave Dispensary, £45; Chelsea Provident Dispensary, £12; City Dispensary, £83; City of London and East London Dispensary, £23; Clapham General and Provident Dispensary, £28; Deptford Medical Mission, £14; Eastern Dispensary, £34; East Dulwich Provident Dispensary, £19; Farringdon General Dispensary, £43; Finsbury Dispensary, £52; Forest Hill Dispensary, £38; Gipsy Hill Provident Dispensary, £4; Hackney Provident Dispensary, £6; Hampstead Provident Dispensary, £43; Holloway and North Islington Dispensary, £62; Islington Dispensary, £43; Kensal Town Provident Dispensary, £9; Kensington Dispensary, £67; Kilburn, Maida Vale, and St. John's Wood Dispensary, £38; Kilburn Provident Medical Institution, £28; London Dispensary, £17; London Medical Mission, Endell Street, £47; Marylebone Medical Mission, nil; Metropolitan Dispensary, £47; Notting Hill Dispensary and Maternity (Provident), £16; Paddington Provident Dispensary, £33; Pimlico Provident Dispensary, (Lupus Street), £15; Portland Town Dispensary, £14; Portobello Road Provident Dispensary, £5; Public Dispensary, £47; Queen Adelaide's Dispensary, £33; Royal General Dispensary, £41; Royal Pimlico Provident Dispensary, £47; Royal South London Dispensary, £52; St. George's and St. James's Dispensary, £47; St. George's, Hanover Square, Provident Dispensary, £38; St. John's Wood and Portland Town Provident Dispensary, £33; St. Marylebone General Dispensary, £43; St. Pancras and Northern Dispensary, £43; South Lambeth, Stockwell, and North Brixton Dispensary, £43; Stamford Hill, Stoke Newington, &c., Dispensary, £52; Tower Hamlets Dispensary, £35; Walworth Provident Dispensary, £10; Wandsworth Common Provident Dispensary, £8; Westbourne Provident Dispensary and Maternity, £14; Western Dispensary, £57; Western General Dispensary, £129; Westminster General Dispensary, £28; Whitechapel Provident Dispensary, £19.

ROYAL NATIONAL HOSPITAL FOR CONSUMPTION, VENTNOR.

